# Prognostic Factors for Leiomyosarcoma with Isolated Metastases to the Lungs: Impact of Metastasectomy

**DOI:** 10.1245/s10434-022-11806-8

**Published:** 2022-05-12

**Authors:** Theresa Stork, Balazs Hegedüs, Wiebke Guder, Rainer Hamacher, Jendrik Hardes, Moritz Kaths, Till Plönes, Christoph Pöttgen, Hans-Ulrich Schildhaus, Arne Streitbürger, Juergen Treckmann, Sebastian Bauer, Clemens Aigner, Stéphane Collaud

**Affiliations:** 1 Department of Thoracic Surgery, Ruhrlandklinik, University of Duisburg-Essen, Essen, Germany; 2 German Cancer Consortium (DKTK), Center Essen, Germany; 3 Department of Tumor Orthopedics and Sarcoma Surgery, University Hospital Essen, University of Duisburg-Essen, Essen, Germany; 4 Department of Oncology, University Hospital Essen, University of Duisburg-Essen, Essen, Germany; 5 Department of General, Visceral, and Transplantation Surgery, University Hospital Essen, University Duisburg-Essen, Essen, Germany; 6 Department of Radiation Oncology, University Hospital Essen, University of Duisburg-Essen, Essen, Germany; 7 Institute of Pathology, University Hospital Essen, University of Duisburg-Essen, Essen, Germany

## Abstract

**Background:**

Leiomyosarcoma (LMS) most frequently metastasizes to the lung. Metastatic LMS is considered incurable. Selected patients may benefit from pulmonary metastasectomy (PM) within multimodal therapy. This study analyzed the prognostic relevance of clinicopathologic factors in these patients.

**Methods:**

Patients with metastatic LMS to the lung treated in our center from 2004 to 2020 were included in this single-center retrospective study. Overall survival (OS), progression-free survival (PFS), and prognostic factors were analyzed.

**Results:**

The study had 64 patients (33 males, 52%) with metastatic LMS to the lung. The 5-year OS was 55% after the diagnosis of pulmonary metastases. Age older than 60 years at the primary tumor diagnosis, primary tumor larger than 70 mm, and five or more lung metastases were associated with poorer OS. Of the 64 patients, 44 underwent PM. The postoperative mortality rate was 0%. The patients selected for PM were younger and had smaller primary tumors, fewer metastases, and metastases that more often were metachronous. Metastasis grade (G1 vs. G2/3) and size (20-mm cutoff) were significant prognostic factors for OS (*p* = 0.05) and PFS (*p* = 0.028) after PM, respectively. The 44 patients who underwent PM had a survival benefit compared with the patients who were selected but did not undergo PM (*n* = 6) and the patients who were not selected for PM (*n* = 14). Three patients (7%) were alive and free of disease at the last follow-up visit respectively 5.5, 9, and 12 years after PM.

**Conclusions:**

For patients with leiomyosarcoma, PM is safe. Despite aggressive multimodal treatment, most patients will experience recurrence and eventually die of their disease. However, a small subgroup of patients could potentially be cured after PM.

Soft tissue sarcomas constitute a heterogeneous group of rare tumors including more than 50 different histologic subtypes.^1^ Leiomyosarcoma (LMS) is one of the most common histologies, accounting for 5–10% of soft tissue sarcomas.^1,2^ Patients most frequently encounter LMS in the fourth to sixth decades of life.^2^ The most frequent sites of primary LMS are the uterus, retroperitoneum, and extremities, but LMS can occur in all body parts.

Despite optimal local therapy, including surgery and radiation in selected cases, metastatic progression is frequent. Metastatic progression is encountered in about 40% of patients during the first 5 years after diagnosis.^3^ Typically, LMS spreads hematogenously, and the lung is the most common organ for metastases.

Pulmonary metastases develop in 25% of patients with LMS.^4^ In a competing risk analysis including 353 patients with LMS from different organs, predictors for distant recurrence were primary tumor size (>10 vs. ≤10 cm: hazard ratio [HR] 2.6, 95% confidence interval [CI] 1.5–4.6; *p* = 0.001) and grade (high vs. low: HR 3.9; 95% CI 1.9–7.8; *p* < 0.001).^5^

In most cases, metastatic LMS is an incurable disease, which usually is treated with first-line doxorubicin-based chemotherapy.^1,6^ Despite the lack of evidence based on randomized clinical trials, pulmonary metastasectomy (PM) usually is offered to selected patients with resectable isolated lung metastases and good performance status.^7^ Indeed, PM is the only potentially curative method for patients with lung metastatic sarcoma.^1,8^

This study examined our isolated lung metastatic LMS cohort and investigated potential prognostic factors.

## Methods

We retrospectively analyzed all patients with metastatic LMS to the lung or lungs only treated at the West German Cancer Center Essen between January 2004 and December 2020. Data were collected from a dedicated sarcoma database, the patients’ electronic documentation system at our center, and all follow-up centers. We compared the two groups of patients: those with and those without PM. The following variables were studied: age, gender, grade of primary tumor and metastases, size of the primary tumor, and time to lung metastasis between primary tumor diagnosis, and appearance of pulmonary metastases, as well as number, size, laterality and timing of pulmonary metastases.

For all the patients who underwent PM, grade, size, and number of metastases were retrieved from pathologic reports. For the patients who did not have PM, size and number of metastases were obtained from chest computed tomography (CT) at the time pulmonary metastases were diagnosed. The institutional ethics committee approved the study (18-7943-BO), and patient consent was waived.

Treatment strategy for all the patients was discussed in an interdisciplinary sarcoma board at each time point during the course of disease relevant for therapeutic decisions. Follow-up evaluation was performed according to our center’s guidelines. Imaging for high-grade sarcoma included a chest/abdomen CT and magnetic resonance imaging (MRI) of the primary region, if required, every 3 months during the first 2 years. In the third year, the interval was prolonged to every 4 to 6 months. From the fourth year, imaging was performed every 6 months up to 5 years. For low-grade sarcoma, MRI was performed every 6 months up to 5 years. Both CT of the chest/abdomen and chest x-ray/abdomen sonography were performed alternating every 6 months. After 5 years, yearly follow-up imaging was discussed on a case-by-case basis.

Patients were eligible for PM if the primary tumor site was completely resected or locally controlled and staging showed the lung to be the only organ system involved with metastatic disease. All lesions were deemed technically resectable. Preoperative evaluation included pulmonary function tests (spirometry, diffusion capacity, and perfusion scintigraphy as well as spiroergometry if necessary) for all the patients. Predicted postoperative values lower than 30% for forced expiratory volume in 1 s (FEV1), carbon monoxide lung diffusion capacity (DLCO), or predicted postoperative maximal aerobic capacity (${{\dot{{\text{V}}}{\text{O}}}}2\max$) of less than 10 ml/(kg/min) were contraindications for PM. Surgical approach was chosen based on location, size, and number of metastases.

Video-assisted thoracoscopic surgery (VATS) wedge resection was performed in case of two or fewer peripheral nodules. An anterolateral muscle-sparing thoracotomy was chosen for the patients with more than two nodules or nodules centrally located. For the patients undergoing thoracotomy, palpation of the lung parenchyma was performed to identify or exclude additional nodules not visible on CT scan. A parenchymal-sparing technique using electrocautery or laser enucleation was preferred. If required, anatomic resections were performed for central metastases. In case of bilateral disease, PM was performed in one or two stages with a 3- to 4-week interval based on tumor load and patient fitness. Lymph node sampling was performed at the surgeon’s discretion.

The distribution of continuous variables was tested by a Shapiro-Wilk normality test. Continuous variables were compared by the unpaired *t* test or the Mann-Whitney test. Fisher’s exact test and the chi-square test were used to compare categorical variables in the PM and non-surgical sub-cohorts. Overall survival (OS) for the whole cohort was calculated from the date of imaging diagnosis of pulmonary metastases until death or the last follow-up visit. For the analysis within the PM sub-cohort, the OS and progression-free survival (PFS) were calculated from the date of PM until progression, death, or the last follow-up visit.

Survival probability was analyzed using Kaplan-Meier curves. Two-stage PM for bilateral disease was considered as one surgical procedure for statistical purposes, and the follow-up period was calculated starting from the first metastasectomy. Time to lung metastasis was defined as the time from diagnosis of the primary tumor to the first radiologic evidence of lung metastases. If pulmonary metastases were detected on initial staging imaging, they were classified as synchronous.

The impact of clinical variables on survival was assessed in univariate analysis by the log-rank test. Median survival, hazard ratios, and 95% confidence intervals were provided. A *p* value of 0.05 or lower was considered significant. Data were analyzed using GraphPad Prism 5 and IBM SPSS 26 statistical software (IBM Corp., Armonk, NY, USA).

## Results

### Prognostic Factors for Leiomyosarcoma Patients with Isolated Lung Metastases

We retrospectively identified 64 patients (33 males, 52%) with lung-only metastatic LMS. The median age at primary tumor diagnosis was 56 years (range, 19–82 years). The most common sites of primary tumor were the uterus (*n* = 19, 30%), followed by the extremities (*n* = 17, 27%), the abdomen/retroperitoneum (*n* = 15, 23%), and others (*n* = 13, 20%).

The median size of the primary tumor was 80 mm (range, 20–200 mm). The patients had a median number of five metastases (range, 1–100). The median size of the metastases was 15.5 mm (range, 3–70 mm).

**Fig. 1:**
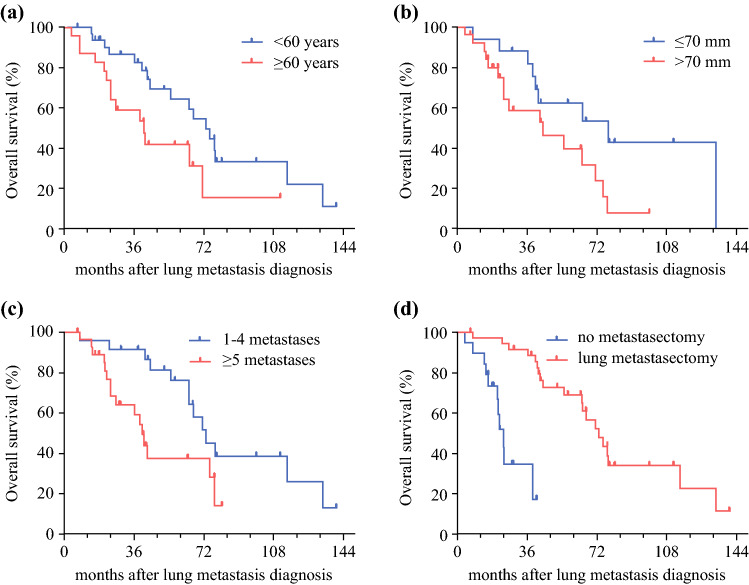
**a** Overall survival after the diagnosis of lung metastasis in leiomyosarcoma patients depending on age at diagnosis of the primary tumor. **b** Size of the primary tumor. **c** Number of metastases. **d** Whether pulmonary metastasectomy was performed or not.

The estimated OS rate from diagnosis of pulmonary metastases was 75% at 3 years and 55% at 5 years. The number of metastases had a significant impact on OS. The patients with five or more metastases had a significantly worse median OS after diagnosis of pulmonary metastases than the patients with fewer than five metastases (40 vs. 73 months; *p* = 0.015; Table 1; Fig. 1). Age older than 60 years at the primary diagnosis and primary tumor larger than 70 mm also were negative prognostic factors (Table 1, Fig. 1A–C).

**Table 1:** Overall survival after the diagnosis of lung metastasis in leiomyosarcoma patients

	Median OS (months)	HR	95% CI	*p* Value
*Gender*				
Female	64.4	1	0.304–0.1344	0.238
Male	71.4	0.64		
*Age at primary (years)*				
≥60	41.1	1	0.182–0.926	**0.032**
<60	73.2	0.41		
*Grade of primary*				
G1	78	1	0.819–5.322	0.123
G2–G3	55	2.09		
*Primary site*				
Other	64.4	1	0.538–2.355	0.754
Gynecologic	71.4	1.125		
*Primary tumor size (mm)*				
≤70	78	1	1.012–5.2	**0.047**
>70	44.4	2.294		
*Lung metastasis*				
Synchronous	24	1	0.193–1.662	0.301
Metachronous	66.7	0.566		
*No. of metastases*				
1–4	73.2	1	1.214–6.053	**0.015**
≤5	40.3	2.711		
*Metastasis size (mm)*				
≤20	66.7	1	0.594–3.023	0.48
>20	64.6	1.34		
*Pulmonary metastasectomy*				
Yes	73.2	1	6.594–80.88	**<0.0001**
nO	24	23.1		

Bold values represent significant *p*-values

*OS* overall survival, *HR* hazard ratio, *CI* confidence interval

### Comparison of Patients With and Without Pulmonary Metastasectomy

The clinicopathologic characteristics of the patients with and without PM were compared (Table 2). The patients who did not undergo PM were significantly older and had larger primary tumors, more lung metastases, and synchronous pulmonary metastases more often than those who underwent PM. The 44 patients who underwent PM had significantly better OS than those without PM (*n* = 20; *p* < 0.001, Fig. 1D). The estimated 3-year survival rate after diagnosis of metastases was 86% for the PM cohort and 35% for the non-surgical cohort.

**Table 2:** Clinicopathologic characteristics of isolated lung metastatic leiomyosarcoma with pulmonary metastasectomy (PM) versus without PM

	Total (*n* = 64)	Pulmonary metastasectomy (*n* = 44)	No pulmonary metastasectomy (*n* = 20)	*p* Value
*Gender*				
Male	33	22	11	0.798
Female	31	22	9	
*Age at primary (years)*				
Mean ± SD	55.8 ± 13.7	52.3 ± 12.8	63.5 ± 12.8	**0.002**
*Grade of primary tumor*				
1	6	4	2	0.1875
2	16	13	3	
3	33	18	15	
*Primary tumor size (cm)*				
Mean ± SD	8.6 ± 4.3	7.5 ± 3.8	10.2 ± 4.7	**0.038**
*Lung metastasis timing*				
Synchronous	11	2	9	**0.0003**
Metachronous	47	37	10	
*Time to metastasis (months)*				
Median (range)	15 (4–242)	26 (4–242)	8 (5–12)	**0.002**
*No. of metastases*				
Median (range)	5 (1–100)	3 (1–34)	17	**<0.0001**
*Metastasis size (mm)*				
Median (range)	16 (3–70)	17 (3–70)	15 (5–55)	0.9839

Bold values represent significant *p*-values

*SD* standard deviation

**Fig. 2:**
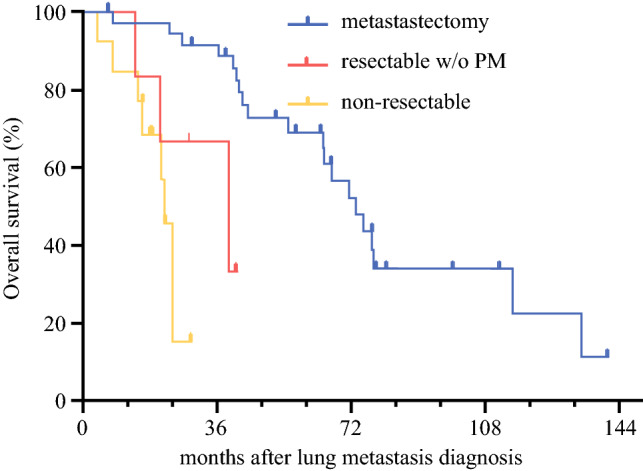
Overall survival comparison of patients who underwent pulmonary metastasectomy (PM) for pulmonary metastasectomy with candidates who did not undergo PM and non-surgical candidates.

The reasons for not performing PM in our cohort were progressive disease under chemotherapy of metastases (*n* = 6, 30%) or the primary tumor (*n* = 1, 5%), the high tumor load (*n* = 6, 30%), and a complete response to chemotherapy (*n* = 1, 5%). For six patients (30%), PM finally was not performed although they were candidates for PM based on clinical chart and CT imaging. The OS for these patients was intermediate between the patients who underwent PM and those who did not. The differences in survival were significant (*p* < 0.001), with a median survival period of 78 months for the patients after PM, 32 months for the PM candidates who did not undergo PM, and 20 months for the non-surgical candidates (Fig. 2).

### Prognostic Factors for Patients with Leiomyosarcoma After PM

Pulmonary metastasectomy was performed for 44 patients (69%). The patients had a median of three metastases (range, 1–34). The median size of the metastases was 17 mm (range, 3–70 mm). The PM was performed via anterolateral thoracotomy (*n* = 41, 93%) or VATS (*n* = 3, 7%). All the patients who underwent VATS PM had one solitary peripherally located metastasis. Lymph node sampling was performed for 23 patients (52%). No lymph node involvement was identified except in one patient (4%), who had a single interlobar positive lymph node. The 90-day postoperative mortality rate was 0%.

Repeat PM was performed for 15 patients (34%). Two PMs were performed for nine patients (60%), three PMs for three patients (20%), and four PMs for two patients (13%). One patient (7%) underwent six PMs.

Of 17 patients (39%) who survived more than 5 years after the first PM, 7 were still alive at the last follow up evaluation. Three of these patients (7%) were free of disease and could be considered as cured respectively 5.5, 9, and 12 years after repeated PM.

**Fig. 3:**
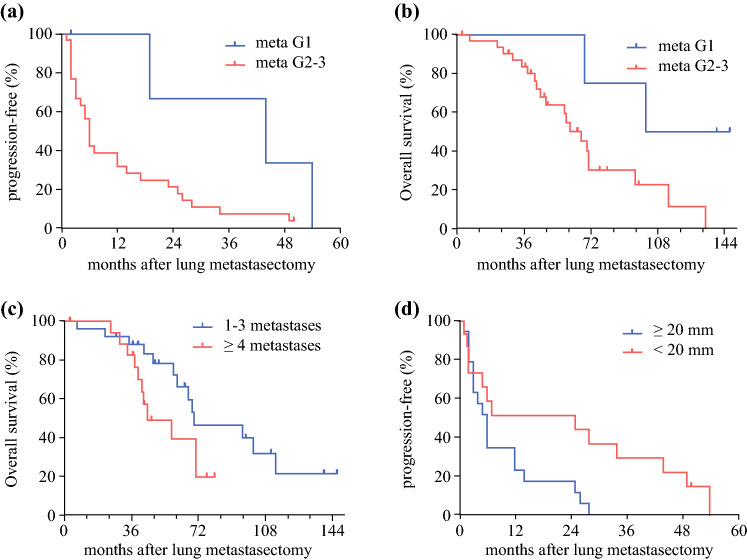
**a** Progression-free and **b** overall survivals after pulmonary metastasectomy depending on grade of metastases. **c** Overall survival after pulmonary metastasectomy depending on the number of metastases. **d** Progression-free survival after pulmonary metastasectomy depending on the size of metastases.

For the patients who underwent PM, grading of metastases was a significant prognostic factor for OS (*p* = 0.05), whereas size of metastases was a significant factor for PFS (*p* = 0.01). Patients with fewer than four lung nodules tended to have a longer OS. Table 3 and Fig. 3 summarize the evaluation of clinical variables as potential prognostic factors for OS and PFS after PM.

**Table 3:** Progression-free and overall survival after the lung metastasectomy in leiomyosarcoma patients

	PFS (months)	HR (95% CI)	*p* Value	OS (months)	HR (95% CI)	*p* Value
*Gender*						
Male	6	0.921	0.81	71	1.031	0.94
Female	12	0.471–1.802		61	0.465–2.289	
*Age at metastasectomy (years)*						
≤55	12	1.187	0.251	67	0.813	0.235
>55	7	0.601–2.418		70	(0.353–1.874)	
*Grade of metastasis*						
G1	44	0.391	0.052^a^	124	0.362	**0.05**
G2–G3	6	0.152–1.008		67	0.131–1	
*Side of metastasis*						
Unilateral	12	0.648	0.244	70	0.559	0.179
Bilateral	6	0.312–1.344		45	0.239–1.306	
*No. of metastases*						
1–3	12	0.602	0.169	70	0.442	0.079
≥4	6	0.292–1.241		45	0.178–1.101	
*Metastasis size (mm)*						
<20	25	2.515	**0.028**	70	1.234	0.648
≥20	6	1.105–5.723		59	0.499–3.047	

Bold values represent significant *p*-values

*PFS* progression-free survival, *HR* hazard ratio, *CI* confidence interval, *OS* overall survival

^a^The Gehan–Breslow–Wilcoxon test results in a *p* value of 0.048.

## Discussion

Among more than 50 different histologic entities of soft tissue sarcomas, LMS is one of the most common. Due to the rarity of soft tissue sarcomas, reports on PM for LMS as a single entity are rare. Most often they are combined with other soft tissue sarcoma entities or with a wide range of tumors of gynecologic origin.^9,10^

In our study, the estimated 5-year survival for all isolated lung metastatic LMS patients was 55%. This compares favorably with reports from the literature with 5-year survivals ranging from 38 to 52%.^8,11,12^ The age at diagnosis, the size and grade of the primary tumor, and the number of metastases had a prognostic impact on OS.

Because randomized clinical trials are not available for PM, we compared the two sub-cohorts of patients who had lung metastatic LMS with or without PM. We found a statistically significant survival benefit for patients who underwent PM. Our results are in line with findings from another report, which showed PM as an independent significant positive prognostic factor for OS with an HR of 0.52 (95% CI 0.38–0.87; *p* = 0.012).^1^ Of course, we should not race to the simplistic conclusion that PM is responsible for this survival benefit because results may be highly distorted by selection bias. Indeed, the exclusion criteria for PM in our study were high tumor load or disease progression under chemotherapy for most patients. In addition, the prognostic profile of both patient cohorts was largely in favor of patients with PM. The patients with PM were younger, had fewer metastases, and had smaller primary tumors. However, the metastasectomy procedure itself could contribute to improved OS.

Importantly, six candidates for PM (and therefore not subjects of initial selection bias) who eventually did not undergo PM also were included in the study. Interestingly, OS for these six patients was intermediate between the cohort of PM candidates who had PM and the cohort of patients not selected for PM, suggesting a potential survival benefit of the PM procedure itself. These results were similar to those for a subgroup analysis of patients with pulmonary metastatic osteosarcoma, who showed better OS and PFS after PM than patients with resectable metastases who did not undergo metastasectomy.^13^ On the contrary, in the subgroup analysis including non-osteosarcoma patients, survivals did not differ between the patients with resected pulmonary metastases and those who underwent systemic treatment alone.^13^

Leiomyosarcomas are considered incurable once metastases are diagnosed. This contrasts with patients who have osteosarcomas, among whom approximately one third of patients with metastatic disease at diagnosis become long-term survivors if they are treated with polychemotherapy and complete surgical resection of all tumors.^14^

Our data strongly underscore the assumption that most patients with LMS will experience recurrence despite aggressive treatment including PM. However, seven patients were still alive more than 5 years after PM. Three of these patients underwent a second PM procedure and were free of disease at the last follow-up visit respectively 5.5, 9, and 12 years after PM. These three patients accounted for 7% of the PM cohort and suggest a potential curative benefit of PM within multimodal therapy in pulmonary metastatic LMS. We believe these data are a valuable reference for advising patients. However, late relapses are not uncommon in LMS, and a longer follow-up period will be helpful to confirm this observation.

Our study did not analyze the impact of chemotherapy on survival. But clearly, effective micrometastases treatment with systemic therapy is the main goal for medical treatment of these patients. Unless a more effective chemotherapeutic regimen is available, long-term survival for patients with LMS will remain unlikely despite PM. The recent LMS-04 trial that combined doxorubicin and trabectedin has yielded the highest overall response rates of any chemotherapeutic combination for LMS to date.^15^ Future studies are needed to determine whether this could increase the number of patients with long-term disease control.

In our surgical cohort, a low grade of pulmonary metastases was significantly associated with longer OS. These findings corroborate earlier studies, in which grading of primary LMS was a strong predictor for distant recurrences (HR 3.9; 95% CI 1.9–7.8).^5^ We also found that the size of pulmonary metastases was a significant predictor for PFS. These results are in line with earlier studies of OS after PM for other soft tissue sarcoma and osteosarcoma cohorts.^16^

Our study had some limitations due to its relatively small sample size, retrospective nature, and incomplete data on primary tumor therapy. However, it is one of the largest studies from the literature focusing on LMS as a single entity within soft tissue sarcoma. Inherent selection bias due to the retrospective study design was thoroughly taken into account, analyzed, and discussed in this report.

## Conclusion

Pulmonary metastasectomy is a safe procedure that can be performed without perioperative mortality for selected patients. Patient selection for PM is at least partially responsible for the improved survival of patients after PM, but our data suggest that the PM procedure itself also might contribute to a survival benefit. Although metastatic LMS is generally considered an incurable disease and most patients with metastatic LMS will experience recurrence despite aggressive treatment including PM, some selected patients could be potentially cured after PM.
